# Comparing robotic ventral transabdominal preperitoneal repair (rvTAPP) and laparoscopic enhanced view totally extraperitoneal repair (eTEP): a multicentre observational study

**DOI:** 10.1007/s11701-025-02443-w

**Published:** 2025-06-12

**Authors:** Johannes Baur, Julian Süsstrunk, Michael Meir, Beat P. Müller, Christoph-Thomas Germer, Christian Jurowich, Daniel Wöhl, Rosita Sortino, Daniel C. Steinemann, Jörg Filser, Fiorenzo Angehrn

**Affiliations:** 1 Department of Visceral Surgery, University Digestive Health Care Center, Clarunis, St. Clara Hospital and University Hospital Basel, Basel, Switzerland; 2 Department of General, Visceral, Transplantation, Vascular and Pediatric Surgery, University Hospital Würzburg, Würzburg, Germany; 3 Department of General, Visceral and Oncologic Surgery, Innklinikum Altötting, Altötting, Germany

**Keywords:** Ventral hernia, Hernia repair, Robotic surgery, Ventral TAPP, ETEP RS

## Abstract

This study compares two emerging minimally invasive techniques—laparoscopic enhanced-view totally extraperitoneal (eTEP) and robotic ventral transabdominal preperitoneal repair (rvTAPP)—for the repair of small and mid-sized ventral hernias. A prospective observational study was conducted using data from the international CROSSFIRE database with patients treated between January 2023 and December 2024. The inclusion criteria were primary and incisional midline ventral hernias ≤ 4 cm, treated with eTEP or rvTAPP. The group comparisons were conducted using propensity score matching, adjusting for differences in body mass index, sex, and hernia defect. A total of 165 patients from three centers were included (120 eTEP, 45 rvTAPP). After propensity score matching, 100 patients were included (57 eTEP and 43 rvTAPP). The pain scores after eTEP and rvTAPP at 2 days postoperatively (2.9 vs. 3.3, *p* = 0.385) and at 6 weeks (2.1 vs. 2.1, *p* = 0.888) were comparable. The mean comprehensive complication index was similar between eTEP and rvTAPP (1.7 vs. 1.5, *p* = 0.561). The operation time was 88.8 min for eTEP and 110.1 min for rvTAPP (*p* = 0.003), whereas rvTAPP involved more teaching procedures (32.6% versus 8.8%; *p* = 0.004). Surgeon workload, as assessed by NASA Task Load Index, was higher in eTEP than rvTAPP (36.3 vs. 17.1; *p* < 0.001), indicating greater physical and mental strain. Both posterior minimally invasive ventral hernia repair techniques—eTEP and rvTAPP—are safe and show comparable pain levels at 2 days and 6 weeks. Robotically assisted ventral TAPP has a longer operative time than eTEP but imposes less workload on the surgeon.

## Introduction

The prevalence of small and mid-sized ventral hernias in the general population can be as high as 43.5%, significantly impacting patients’ quality of life [1]. Despite being a common condition and a frequently encountered surgical issue, the optimal surgical approach for ventral hernia repair remains debated. The current guidelines, endorsed by the European and American Hernia Societies in 2019, recommend the open preperitoneal mesh technique for repairing umbilical and epigastric hernias [2]. Despite the growing shift toward minimally invasive surgery, laparoscopic intraperitoneal onlay mesh (IPOM) repair—once the dominant method—has seen a decline in popularity, giving way to open mesh-reinforced repairs or minimally invasive extraperitoneal techniques [3]. However, open repair is associated with higher risks of surgical site infections and long-term complications such as hernia recurrence, especially in patients with common risk factors like obesity and type 2 diabetes mellitus [4, 5]. Recent systematic reviews have examined 13 studies on newly developed techniques such as enhanced-view totally extraperitoneal repair (eTEP) and 27 studies on various anterior and posterior minimally invasive mesh-reinforced methods [6, 7]. The key findings suggest that while these new techniques are generally safe and feasible, anterior approaches involving subcutaneous mesh placement are associated with high rates of seroma and infection. Conversely, posterior approaches, such as eTEP with mesh placement in the retrorectus space, have shown promising early results compared to laparoscopic IPOM, with patients experiencing lower postoperative pain [8]. However, the downside of eTEP ventral hernia repair lies in its steep learning curve and, in rare cases, the occurrence of complications such as bleeding or intraparietal hernias, which are related to the excessive dissection of the abdominal wall [9].

With increased use of robotic assistance in ventral hernia repair, using the preperitoneal space for mesh placement in small or medium primary and incisional hernias has come into focus. The techniques such as the robotic ventral transabdominal preperitoneal repair (rvTAPP) offer the possibility of extraperitoneal mesh placement without the need of excessive dissection to the posterior rectus sheath. However, so far there is only limited data available if this may lead to an improved short-term outcome in ventral hernia repair. The purpose of this study was to determine whether rvTAPP has improved short-term outcomes in terms of postoperative pain and complications compared to eTEP.

## Methods

### Design and subjects

This prospective observational study uses data from the international CROSSFIRE (Multi**C**entre Inte**R**national Pr**OS**pective Databa**S**e **F**or Ventral Hern**I**a **RE**pair) database. This database along with collaborating surgeons is focused on investigating minimally invasive ventral hernia repair techniques. It includes data from three specialized hernia centers (one in Switzerland and two in Germany) of which one is specialized in robotic ventral hernia repair. The study was approved by the local ethics committee (EKNZ 2024-01463), and all patients gave written informed consent for participation. The study adheres to the Strengthening the Reporting of Observational Studies in Epidemiology (STROBE) guidelines [10].

The inclusion criteria for this analysis from the CROSSFIRE database were as follows: minimally invasive repair of ventral hernia using laparoscopic eTEP or rvTAPP between January 2023 and December 2024 in patients with primary or incisional midline ventral hernias measuring 4 cm or less in diameter. No predefined exclusion criteria were applied.

### Preoperative investigations and surgical procedures

**Fig. 1:**
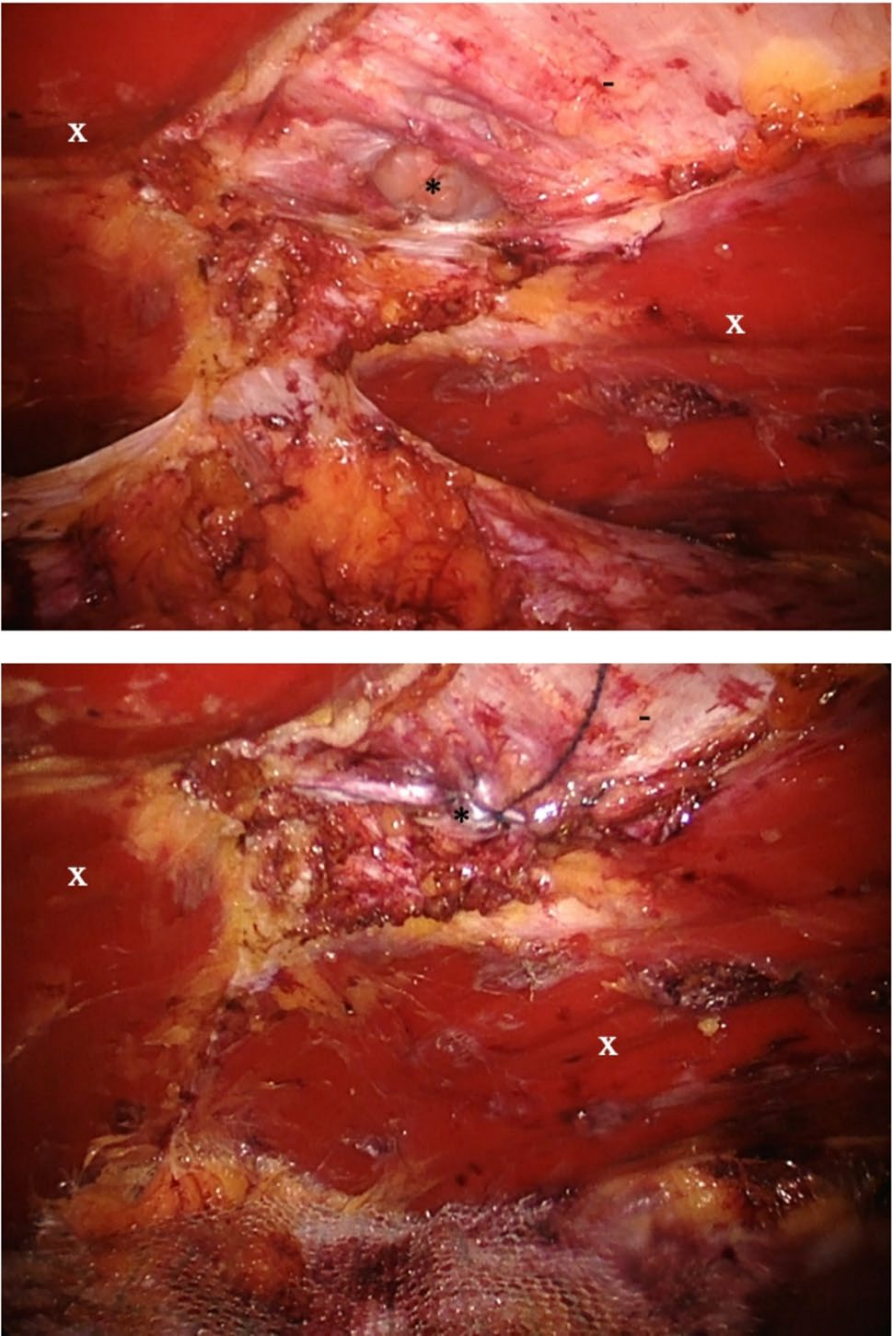
Above: Intraoperative view in enhanced view totally extraperitoneal plasty (eTEP) after nearly complete dissection of both retromuscular planes, linea alba and hernia orifice, seen from cranially. Below: Intraoperative view after complete dissection of retromuscular plane, closure of hernia defect and mesh insertion in retromuscular position. x = rectus muscles, * = hernia orifice

**Fig. 2:**
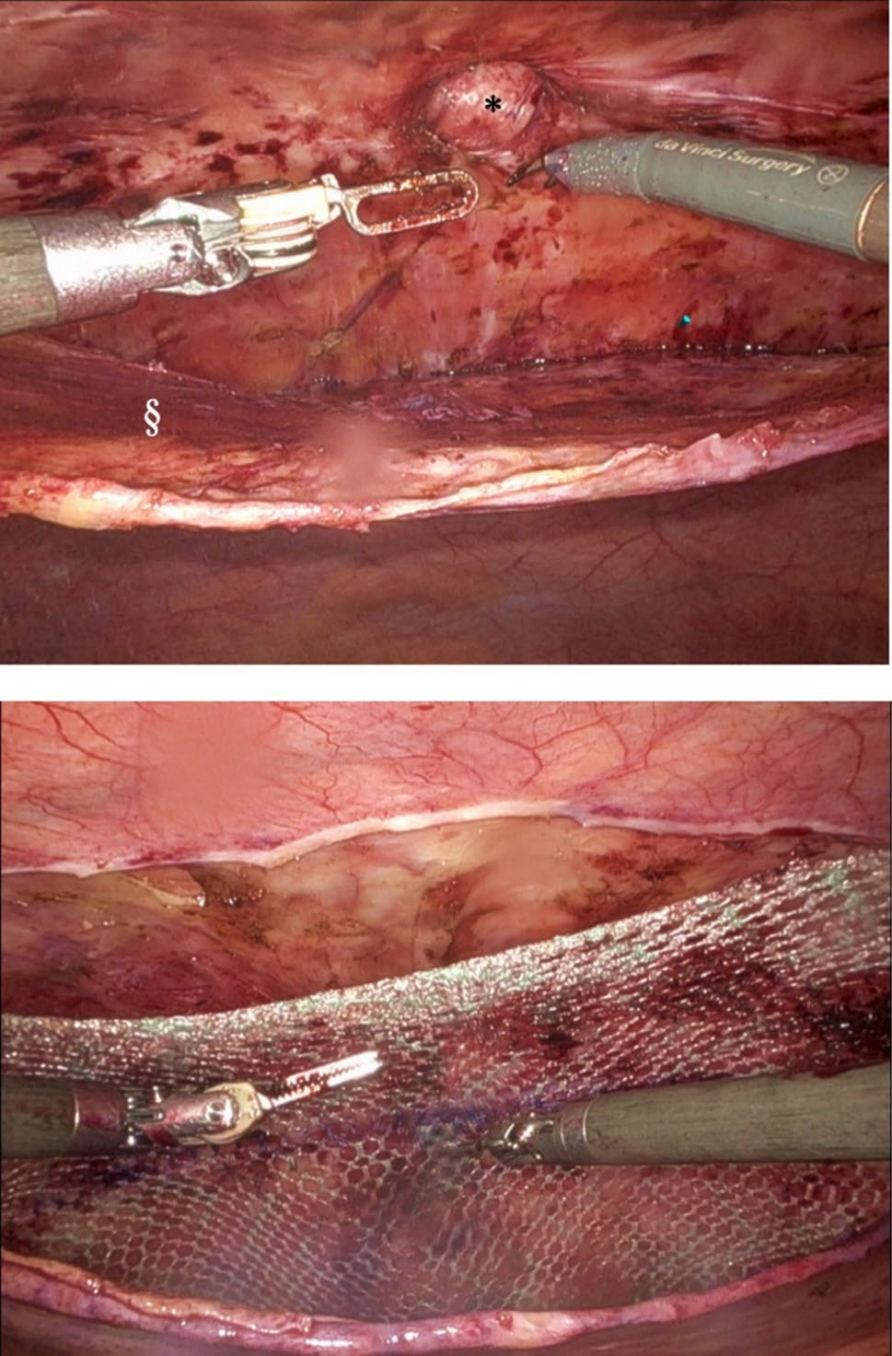
Above: Intraoperative view in robotic ventral transabdominal preperitoneal repair (rvTAPP) after dissection of the preperitoneal flap and during reduction of the hernia sac. Below: Intraoperative view after hernia defect closure and mesh insertion in the preperitoneal plane. § = mobilized peritoneum, * = hernia orifice

Preoperative evaluation included clinical examination of the abdominal wall to assess for hernias and divarication of the rectus muscles. Ultrasound or computed tomography was performed when deemed necessary by the consulting surgeon. All procedures were conducted under general anesthesia, with single-shot intravenous antibiotic prophylaxis 30–60 min prior to surgical incision and with local anesthetics applied at the port sites. For eTEP, three left lateral ports were used to access and dissect the retromuscular space. Superior or inferior crossover was performed as described by Belyansky et al. to access the contralateral retromuscular space [11]. The hernia content was subsequently reduced, and posterior peritoneal defects, if present, were closed using resorbable sutures. The hernia defect was then closed with running absorbable barbed sutures, followed by placement of a non-absorbable retromuscular mesh. In rvTAPP, three robotic 8 mm trocars were inserted in the left abdomen, and the hernia content was reduced. The preperitoneal space was accessed 5 to 8 cm lateral to the midline, with the peritoneum and preperitoneal fat separated from the posterior rectus sheath and the linea alba. The hernia sac and preperitoneal fat were reduced from the hernia orifice. The hernia defect was closed with running absorbable barbed sutures, and a preperitoneal non-absorbable mesh was placed. The lateral peritoneal incision was closed using absorbable running barbed sutures. The detailed data on hernia defect closure and mesh fixation were documented and are reported. Figures 1 and 2 illustrate key steps for both procedures.

After both procedures, standard analgesics (paracetamol and non-steroidal anti-inflammatory drugs) and deep vein thrombosis prophylaxis were administered during inpatient treatment and the same analgesic were given on demand at discharge. The patients were followed up in outpatient clinic 6 weeks postoperatively and will be contacted to assess for recurrence after 1 and 5 years.

### Outcome measures

The primary outcome was pain on a numeric rating scale (NRS, 0 = no pain, 10 = maximum pain) at 2 days and 6 weeks postoperatively.

The secondary outcomes included the incidence of postoperative complications determined by the comprehensive complication index (CCI, 0 = no complications, 100 = death of the patient), length of stay, intraoperative data such as method of hernia defect closure, mesh fixation, defect area, mesh size, mesh to defect ratio, operation time, and the surgeons task load measured using the National Aeronautics and Space Administration Task Load Index (NASA-TLX) [12, 13]. NASA-TLX measures mental, physical and temporal demand as well as performance, effort and frustration, each dimension scales from 0 to 100 points (0 indicating low demand/good performance and 100 indicating maximum demand/worst performance).

### Statistical analysis

To control for potential confounding variables, we employed propensity score matching. The propensity scores were estimated using a logistic regression model, where the rvTAPP group was regressed on body mass index (BMI), sex, and hernia defect area. We performed nearest neighbor matching with a ratio of 2:1 (two eTEP for each rvTAPP subject) using R. A caliber of 0.3 standard deviations of the propensity score was applied to ensure the quality of matches. Due to the availability of suitable matches, the eTEP group did not end up with exactly twice the number of patients as the rvTAPP group. Covariate balance after matching was assessed and found to be satisfactory

Continuous variables were reported as mean (standard deviation), and categorical variables as count (percentage). The statistical comparisons between groups were performed using *t*-tests for continuous variables and Fisher’s exact tests for categorical variables.

Statistical analyses were performed using the RStudio 2024.12.0.

## Results

### Patient and hernia characteristics

A total of 165 patients in the CROSSFIRE database met the inclusion criteria for this study (120 eTEP and 45 rvTAPP). After propensity score matching, 57 patients were included in the eTEP group and 43 to the rvTAPP group. After propensity score matching, there were no significant differences in the relevant baseline characteristics between the eTEP and rvTAPP groups (female sex: 21.1% vs. 30.2%, body mass index: 29.4 vs. 27.9 kg/m^2^, age: 55.1 vs. 53.4 years, primary hernia 71.9% vs. 71.4%, and hernia defect area: 3.6 vs. 3.2 cm^2^). The rate of comorbidities was comparable between the groups. Detailed data on patient and hernia characteristics are displayed in Table 1.

**Table 1:**
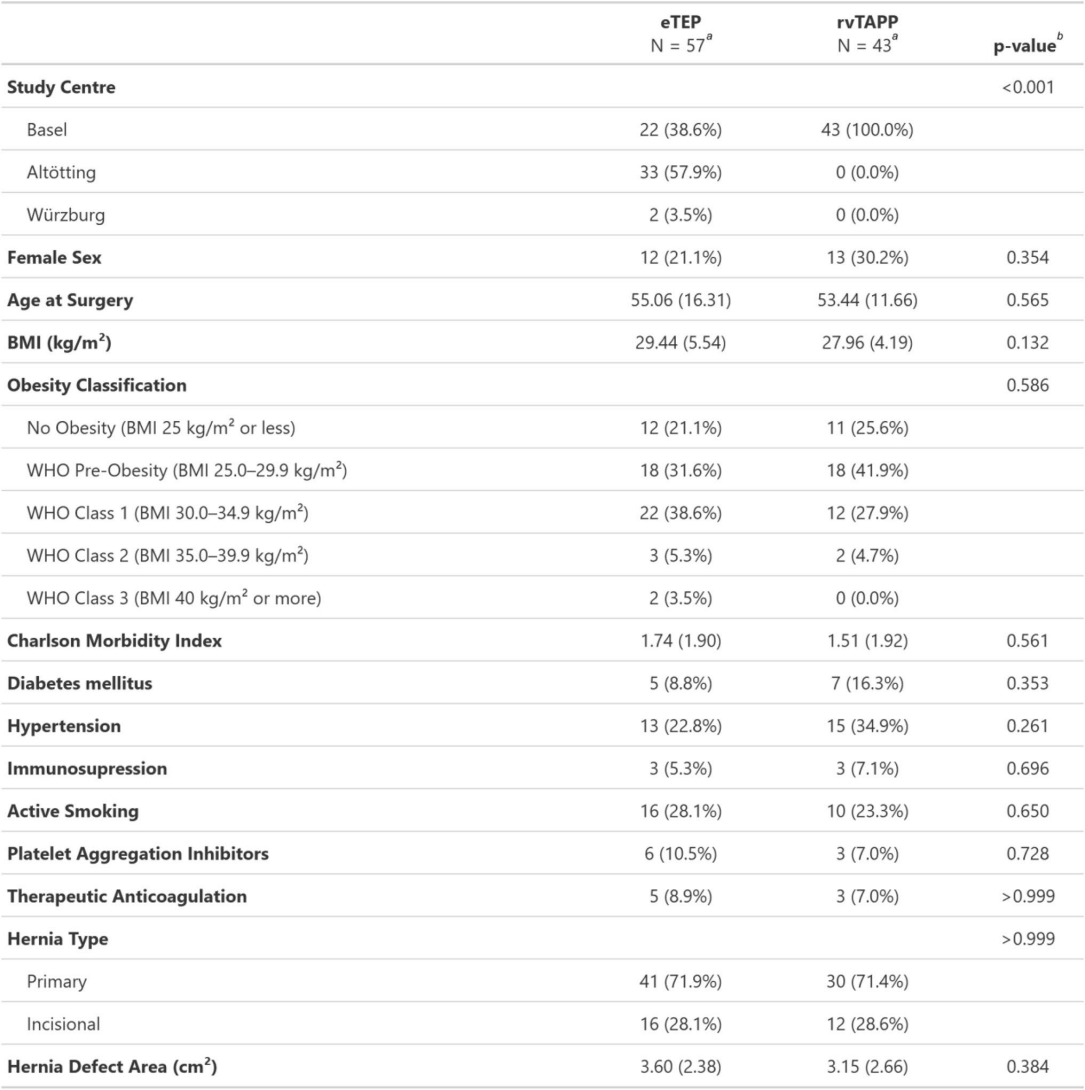
Baseline characteristics

*BMI* Body Mass index, *WHO* World Health organisation

^a^*n* (%); Mean (SD)

^b^Fisher’s exact test; Welch Two Sample *t*-test

### Procedural data

The mean (± standard deviation = SD) operation time was 88.8 ± 26.5 min for eTEP and 110.1 ± 40.1 min for rvTAPP (*p* = 0.003). More teaching procedures were performed in the rvTAPP group (32.6% vs. 8.8%, *p* = 0.004). The hernia defect closure rate was comparable between groups (96% for eTEP vs. 98% for rvTAPP, *p* > 0.999). However, the methods of closure differed, with more longitudinal defect closures performed in the eTEP group (42.1% vs. 11.6%) and more transverse defect closures in rvTAPP group (54.5% vs. 86,0%). The mesh types were similar between groups, but mesh fixation method differed significantly. In the eTEP group, 61.4% of patients received no mesh fixation and 36.8% received cyanoacrylate glue fixation. In contrast, 81.4% of the patients in rvTAPP group received sutured mesh fixation (*p* < 0.001). Surface of the meshes was higher in eTEP group (303.3 vs. 181.1 cm^2^, *p* < 0.001). However, the mesh-defect ratio was comparable between groups.

The concomitant hernial orifices of the umbilical and supraumbilical linea alba were detected in 38.0 % of the patients and this rate did not differ between the two study groups (35.1 % vs. 41.9 %, *p* = 0.537). Detailed procedure-specific data are displayed in Table 2.

**Table 2:**
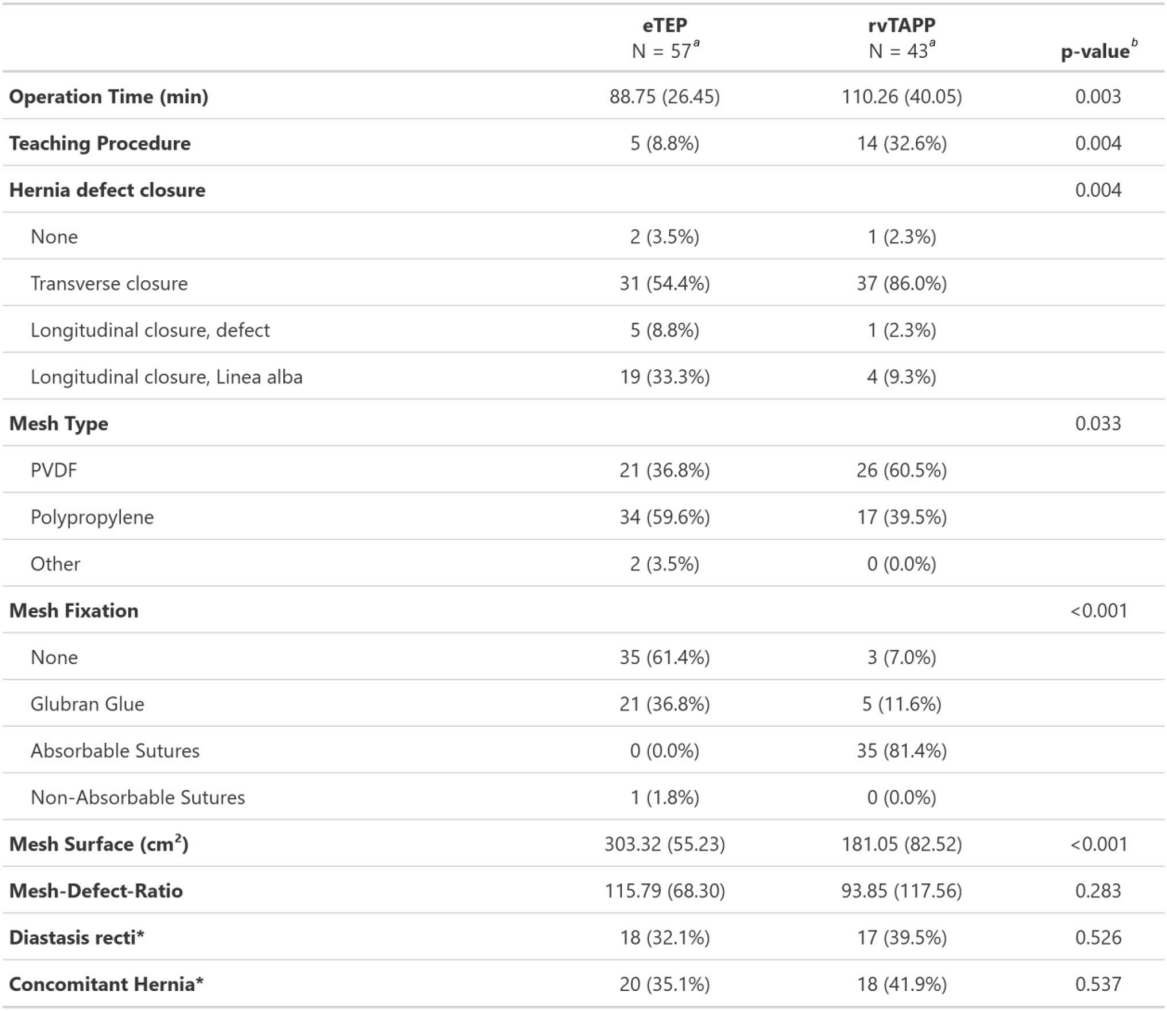
Procedural data

^*^In 48.6% of cases with diastasis recti, a concomitant midline hernia was found

^a^Mean (SD); *n* (%)

^b^Welch Two Sample *t*-test; Fisher’s exact test

Data on surgeon workload, as measured by the NASA TLX, showed higher scores for all subscales in eTEP group except the subscale for “Performance”, indicating a higher mental and physical workload and greater mental distress compared to rvTAPP. Table 3 displays detailed data on the NASA TLX.

**Table 3:**
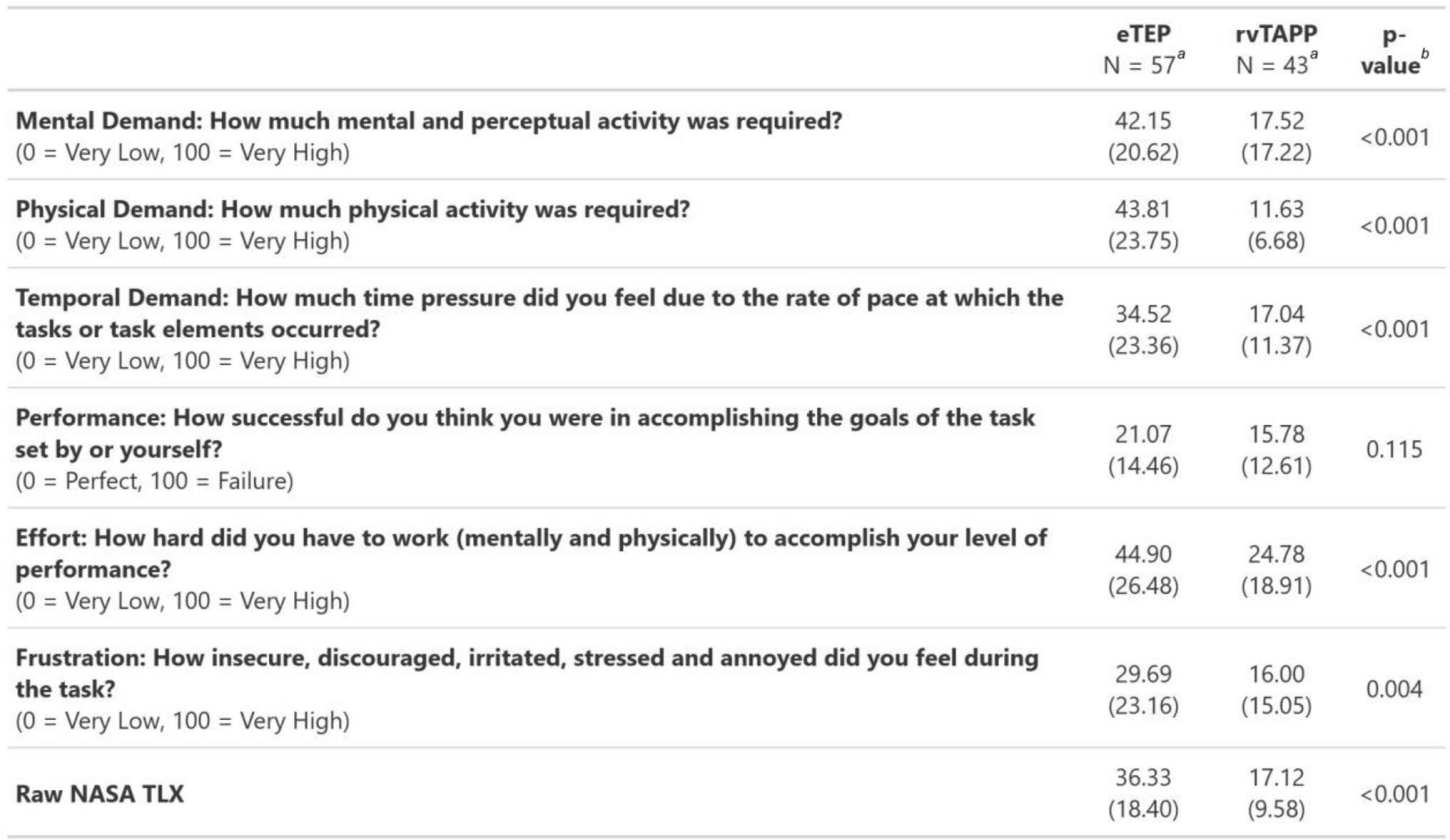
NASA TLX

^a^Mean (SD)

^b^Welch Two Sample t-test

### Perioperative outcomes

The mean (± SD) pain score on a NRS at day 2 (2.9 ± 1.5 vs. 3.3 ± 1.9, p = 0.385) and at week 6 after surgery (2.1 ± 1.7 vs. 2.1 ± 2.4, *p* = 0.888) showed no difference between eTEP and rvTAPP. In rvTAPP, 15% of patients had a VAS greater than 4/10 6 weeks after the procedure compared to 4.1% in eTEP (*p* = 0.133). The clinical follow-up rate was 100% after 6 weeks, whereas NRS data were available in 89% of patients.

The mean (± SD) length of stay after eTEP and rvTAPP was 2.2 ± 1.8 days and 2 ± 0.9 days, respectively (*p* = 0.473).

Two patients (3.5%) in the eTEP group and three patients (7.0%) in the rvTAPP group experienced one or more complications (*p*>0.999). There was 1 operative revision and one radiologic intervention following eTEP. One patient experienced an intraparietal hernia with consecutive ileus due to a herniated small bowel loop through a peritoneal defect between the two posterior rectus sheaths. The complication was addressed laparoscopically with reposition of dislocated bowel structures and replacing the retromuscular mesh by an intraperitoneal onlay mesh. The same patient suffered from previously undiagnosed liver cirrhosis and had an additional bleeding episode from esophageal varices postoperatively requiring endoscopic ligation. The remaining complications (one patient with hematoma after eTEP and two patients with hematomas and one patient with dysesthesia after rvTAPP) were minor and did not necessitate any surgical or endoscopic intervention.

The mean (± SD) CCI was comparable between eTEP (1.7 ± 1.9) and rvTAPP (1.5 ± 1.9, *p* = 0.561). Detailed data on perioperative outcomes is shown in Table 4.

**Table 4:**
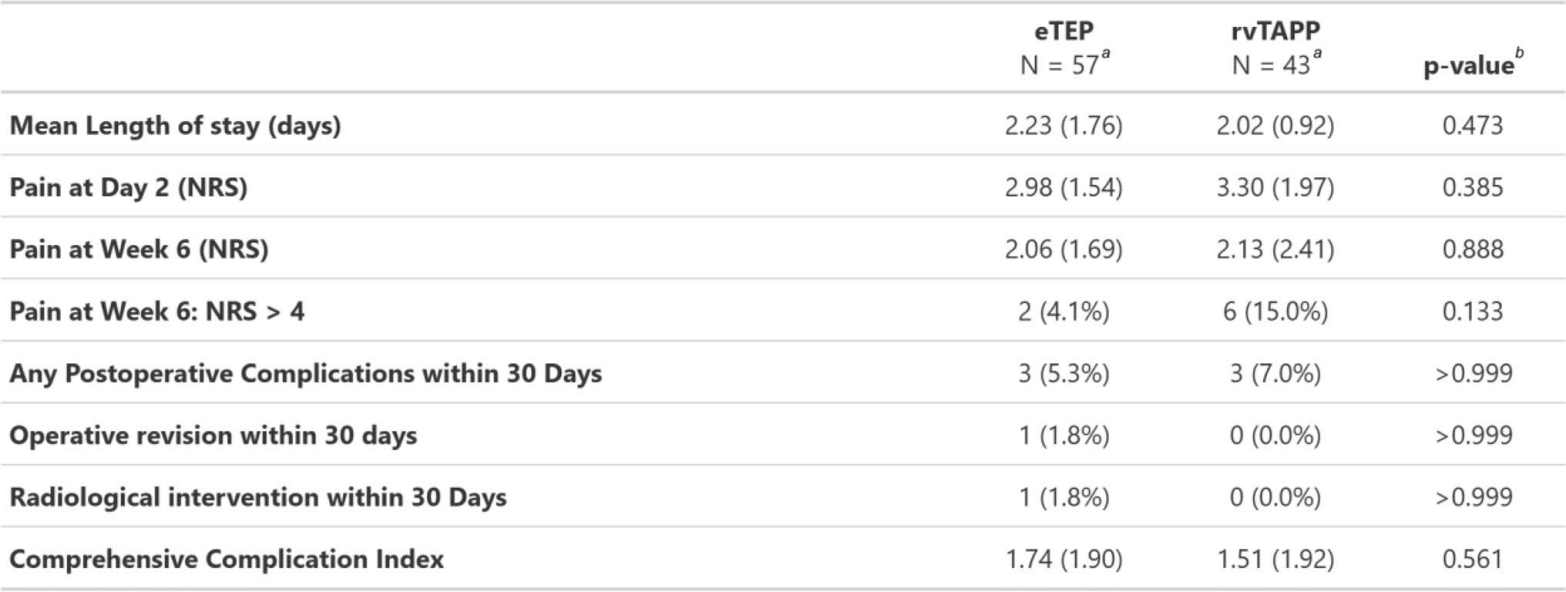
Perioperative outcomes

*NRS* Numeric Rating Scale, 0 = no pain, 10 = Maximum pain

^a^Mean (SD); *n* (%)

^b^Welch Two Sample t-test; Fisher’s exact test

## Discussion

The results of this propensity score matched analysis did not show superiority of rvTAPP compared to eTEP in the treatment of small to medium sized ventral hernias in the short-term. There was no difference in pain levels 2 days and 6 weeks postoperatively between the two groups. Yet not statistically significant, there was a trend towards higher pain scores with NRS of 4 or more after 6 weeks in patients undergoing rvTAPP. This difference might be attributed to the sutures used for mesh fixation and peritoneal closure in the rvTAPP group. In eTEP, there were significantly more cases where no or non-traumatic mesh fixation (cyanoacrylate glue = glubran 2) was applied.

One patient needed surgical and endoscopic intervention. Surgical revision was needed due to an intraparietal hernia between the two posterior rectus sheaths. Although this kind of complication is rare, it is only reported after eTEP in the literature and not after rvTAPP. However, the differences in the need for surgical or non-surgical intervention were not statistically significant and there was also no difference in comprehensive complication index between the two groups. The debate whether the severity of complications may be higher compared to open approaches remains open [14].

The robotically assisted procedure had statistically significant longer operative times which might be due to the fact, that one third were teaching procedures. Among highly trained robotic hernia surgeons, the operative times for rvTAPP are as low or even lower compared to our eTEP group [15]. However, this implicates that the use of dual-console robotic systems in surgical training enhances the education and increases the frequency of hands-on participation in minimally invasive surgical procedures for surgical residents.

Both eTEP and rvTAPP allow for the placement of a large, well-positioned mesh resulting in a high mesh-defect ratio, which not only reinforces the hernia site but also addresses areas of rectus muscle divarication—a known risk factor for future hernia formation[16].

However, the mesh surface area was statistically larger in the eTEP group compared to the rvTAPP group, indicating that the retrorectus space provides a more extensive area for mesh placement. Due to the high rate of diastasis recti and clinically inapparent concomitant hernias of the umbilical and supraumbilical linea alba in 38% of all cases, in our view, large meshes covering the supraumbilical linea alba are essential in preventing hernia recurrence. Yet it remains questionable if the larger meshes in the eTEP group may lead to an improved recurrence rate as there are no significant differences in mesh-defect-ratio between the two study groups.

Surgeon workload, as measured by the NASA-TLX, was higher for eTEP across all sub-scores except for the evaluation of overall performance. This suggests that while both techniques are capable of achieving satisfactory results for the surgeon, eTEP imposes greater physical and mental demands on the surgeon compared to rvTAPP. Retromuscular access, port placement, and hernia dissection are likely more challenging in eTEP explaining the difference in subjective workload. A robotic system, in contrast, allows the surgeon to work more ergonomically, hernia defect closure is technically less demanding due to the additional articulation in robotic instruments and establishment of intraabdominal access is technically less demanding.

This study has some limitations, especially missing long term results regarding recurrence and quality of life after these procedures. So far, the literature provides limited information regarding this issue. Kudsi et al. showed a trend towards a lower rate of patients free from recurrence after rvTAPP compared to robotic retrorectus mesh placement (93.3% vs. 98.2%: *p* = 0.3; follow-up duration up to 5 years).[15] However, hernias with a diameter of 4 cm or more were also treated with rvTAPP in this study, resulting in a smaller mesh-to-defect ratio compared to our rvTAPP-treated group. Therefore, the results of this study cannot be directly applied to our study cohort.

Additionally, a cost analysis for the two techniques is lacking. However, it can be assumed that conventional laparoscopic eTEP is less expensive than rvTAPP or IPOM, as it does not require costly robotic equipment or expensive coated meshes and fixation devices. However, compared to laparoscopic IPOM, rvTAPP may be only slightly more expensive or even cost-neutral, as the costs for robotic devices are offset using cheaper meshes and fixation methods [17].

## Conclusion

Both posterior minimally invasive ventral hernia repair techniques—eTEP and rvTAPP—are safe and exhibit comparable pain levels at 2 days and 6 weeks post-operation. Robotically assisted ventral TAPP has a longer operative time than eTEP, likely due to a higher frequency of teaching procedures in the rvTAPP group. However, rvTAPP imposes less workload on the surgeon.

## Data Availability

Data is available upon request.
